# The Intelligent Attitude: What Is Missing from Intelligence Tests

**DOI:** 10.3390/jintelligence10040116

**Published:** 2022-12-01

**Authors:** Robert J. Sternberg

**Affiliations:** Department of Psychology, Cornell University, Ithaca, NY 14853, USA; rjs487@cornell.edu

**Keywords:** intelligence, creativity, wisdom, personality, openness, cognitive style, attitude, disposition, ability, critical thinking

## Abstract

Intelligence, like creativity and wisdom, has an attitudinal component as well as an ability-based one. The attitudinal component is at least as important as the ability-based one. Theories of intelligence, in ignoring the attitudinal component of intelligence, have failed to account fully or accurately for why so many people who have relatively high levels of intelligence as an ability fail fully to deploy their ability, especially toward positive ends. The article reviews the need to view intelligence as comprising an attitude as well as an ability, and surveys reasons why people’s lack of an intelligent attitude hinders their deployment of intelligence. Suggestions are made for how things could change in a positive way.

## 1. Introduction

Intelligence tests, as they exist today, have a long history, going back at least to the Binet-Simon intelligence tests of the turn of the 20th century (Binet and Simon 1916). Intelligence tests today, more and more, are based loosely on Carroll’s (1993) integrative psychometric theory of intelligence or a variant of it, such as CHC theory (McGrew 2005). However, historically, many of the tests have been largely atheoretical, or alternatively, have measured some combination of the abilities posited by Thurstone’s (1938) theory of primary mental abilities, including measures of a variety of skills, such as verbal comprehension and verbal fluency, quantitative skills, inductive reasoning skills, memory skills, spatial-relations, and perceptual-speed skills.

There are also various proxies for intelligence tests, such as the SAT, ACT, and GRE, which are used in college and university admissions in the United States and elsewhere. All of these measures can be considered to be proxies for the measurement of what Sackett et al. (2020) and others have referred to as general mental ability (GMA). Indeed, the proxies correlate about as highly with tests of intelligence as those tests correlate with each other (Frey and Detterman 2004; Koenig et al. 2008). 

In recent years, some theorists have recommended that broader sets of skills be incorporated into theories and tests of intelligence. For example, Gardner (1983, 2011) suggested that intelligence is multiple, and that tests of multiple intelligences ideally should include all eight of his proposed multiple intelligences: linguistic, logical-mathematical, spatial, bodily-kinesthetic, naturalist, musical, interpersonal, and intrapersonal. Sternberg (1985, 1997a) has suggested that tests include measures not only of knowledge and abstract-analytical skills, but also of creative and practical skills. In an article on musical intelligence, Sternberg (2020b) proposed that his theory could be integrated with Gardner’s, in the sense that each of Gardner’s eight domains involves a combination of creative, analytical, and practical processing. Both Gardner and Sternberg have proposed various kinds of tests that could be used to measure the skills encompassed by their theories, although none of these tests is standardized (e.g., Gardner et al. 1998; Sternberg 2010). There are also tests of specific kinds of intelligence, such as emotional, social, practical, and other kinds of intelligence (see essays in Sternberg 2020a).

## 2. An Attitudinal Aspect of Intelligence

The argument of this article is that intelligence comprises not only a set of abilities, but also a set of attitudes—that it has both ability-based and attitudinally based components. All of the “big three” of human abilities-research—intelligence, creativity, and wisdom--have components of both abilities and attitudes. I define an “ability” here as a developed cognitive capacity that can be modified with instruction and effort and an “attitude” as a developed mindset or approach toward something that is capable of change (see further definitions, e.g., in Banaji and Heiphetz 2010; Merriam-Webster n.d.; Rajecki 1990; Zanna and Rempel 2008). An individual can have an ability, but without an attitude toward using that ability, the ability may remain latent and thus unused, or at best, underutilized.

Research on attitudes went through an explosion of interest in the 1960s, when the cognitive approach to social-psychological phenomena was just forming. This work, although not directly on intelligence, adumbrated the current essay on intelligent attitudes, that is, attitudes toward the deployment of one’s intelligence as a set of abilities. 

If there was a seminal work, it was probably that of Hovland et al. (1953). Hovland and his colleagues were interested in factors that lead to opinion change. Source credibility was found to be highly important in inducing opinion change, somewhat independent of content. These investigators found that opinion leaders who desire to change the opinions of others, but try to hide this desire, often are less effective because listeners may regard them with suspicion. The opinion leaders may do better to state their goal but then try to show why their position is the one listeners should adopt. However, it later turned out that there was a moderator variable. Mills and Aronson (1965) subsequently discovered that if the communicator was physically attractive, they were more likely to be persuasive if they announced their intentions in advance; but if they were unattractive, stating an intention had no effect on opinion change. Kelley and Volkart (1952) found, about the same time as the early Hovland work, that group-induced attitudes are very resistant to change—a phenomenon we see in today’s polarization of the US electorate and of political leaders. 

Janis (1972) later found that even highly educated and presumably intelligent people, including political leaders, often are ineffective because of the attitudes they hold toward how a group should function. That is, their attitude toward their work result in what, in the context of this article, could be called an attitudinal suppression of intelligent functioning. Janis, in particular, studied major foreign-policy decisions made by top leaders within governments. In particular, groups were and still are susceptible to groupthink: (a) illusions of unanimity even when there are disagreements within the group; (b) beliefs that are considered to be true and thus beyond question; (c) rationalizations, whereby the leaders engage in tortuous and tortured critical thinking to reach the conclusion they want to reach; (d) stereotyping, whereby groups are viewed through a single and often distorted lens; (e) formation of often self-appointed mindguards, who ensure conformity within the group; (f) illusions of invulnerability, whereby group members believe that they are beyond reasonable standards of accountability for their decisions and actions; (g) and extreme pressure on those who disagree either to change their position and agree, or else exit the group. 

Rhodes and Wood (1992) looked directly at effects of intelligence on persuasibility. They found, in reviewing the literature, that general intelligence is related to susceptibility to influence. In particular, less intelligent people are more easily influenced, as are those with moderate self-esteem. People with high self-esteem tend to resist influence attempts, and people with low self-esteem often do not understand the message that is supposed to influence them. However, everyone is at least somewhat susceptible to being propagandized and influenced, as Milgram ([1974] 2009) showed in his studies of obedience and as Pratkanis (2001) demonstrated in his work on propaganda.

The “big three” of human-abilities research, mentioned earlier, might be viewed as intelligence, plus creativity and wisdom (e.g., Sternberg 2003; Sternberg et al., forthcoming). Their interrelationship has never been entirely clear, and indeed, a whole recent book has been devoted to elucidating this complex relationship (Sternberg et al., forthcoming). The assumption in much of the theorizing about the constructs—indeed, almost all of it—has been on intelligence as a cognitive ability. However, is it solely an ability?

Tests of targeted kinds of intelligence have been developed that are both ability-based and also attitudinal, for example, for emotional intelligence (see Rivers et al. 2020) and cultural intelligence (see Van Dyne et al. 2008). The general finding has been that attitudinal measures, usually typical-performance measures based on self-report, show little or no correlation with ability-based measures, usually maximum-performance measures (e.g., Rivers et al. 2020; Sternberg et al. 2021, 2022). In contrast to measures of general intelligence, moreover, are measures of creativity and wisdom, which generally are either ability-based or attitudinally based. The correlations between the two types of measures are low (Glück 2022; Plucker et al. 2021). Yet, both types of measures can yield real-world correlates (see, e.g., Ang et al. 2020; Van Dyne et al. 2008). Is it possible that experience with the measurement of creativity and wisdom, as well as measurement of specific kinds of intelligence (such as emotional and cultural), could contain lessons for the measurement of intelligence in general—namely, that there is, in addition to an ability-based component of intelligence, an attitudinal component that theories and tests largely have neglected?

Sometimes, it has seemed that the field of intelligence, and indeed, parts of psychology more generally, have become so immersed in measurement that they have not always kept track of the forest for the trees. Measurement has often seemed to be more an end than a means: Small effects take on a life of their own, often independent of all the confoundings to which they are subject (see, e.g., Smedslund 2016; Uher 2021a, 2021b). This can happen in any research, including, of course, the present exemplar.

Measures of intelligence or anything else as an attitude almost inevitably involve self-report. The problems with self-report measures of psychological constructs are well-known and are covered elsewhere (e.g., Lundmann and Villadsen 2016; Rosenbaum and Valsiner 2011; Sternberg et al. 2021, 2022; Uher 2018; Wagoner and Valsiner 2005; Williamson and Hoggart 2005). First, the measures are susceptible to lying: Anyone can say anything they want about what they are like or aspire to be like. Second, the measures are susceptible to self-deception. The fact that people have a certain “implicit theory” about what they are like does not mean they are like that. Indeed, the Dunning-Kruger effect (Kruger and Dunning 1999) suggests that often the least competent people view themselves as the most competent. Third, people use Likert or other related scales in different ways. One person’s 6 (on a 1–9) scale might be another person’s 7 or even 8. Moreover, some people tend to cluster their results near the center of the scale, others to use extremes more often, and still others to have a more even spread of ratings. Fourth, the scales tend to be domain-general: They typically characterize the way a person supposedly is, in general, as opposed to the way they are in specific situations. However, at least some attributes, especially creativity and wisdom, appear to be somewhat domain- or situation-specific (Baer 2015; Grossmann 2022). Fifth, typical-performance measures are susceptible to halo effects: If people see themselves as a certain way, they may tend to answer most or all questions in a way that supports that perception.

Although self-report measures are limited as bases of measurement of human attributes, so are all measures. The different kinds of measures simply have different limitations and thus different sources of error. For example, maximum-performance task-based measures are limited because (a) there is no guarantee that what people do on a hypothetical task will correspond to what they will do on a real-world task, (b) the problems typically can only minimally measure the breadth of the behavioral or other domain of interest, (c) scoring often is challenging and potentially subjective, (d) if the tasks are complex, there may not be enough of them to gain sufficient reliability and validity, and (e) the tasks may not even correspond to the real-world that they are supposed to correspond to—for example, they may lack the high stakes, ideological resonance, or emotional freight that typically accompany high-stakes real-world problems (for related points, see Molenaar 2008; Molenaar and Campbell 2009; Richters 2021; Salvatore and Valsiner 2010; Uher 2021a, 2021b).

The conclusion some have drawn is that choosing between typical- and maximum-performance measures of psychological constructs may present a false dichotomy. Both typical-performance and maximum-performance measures have their place in the measurements of certain psychological constructs (e.g., Rivers et al. 2020; Sternberg et al. 2022). The two kinds of measures simply measure different aspects of a psychological phenomenon. For some attributes, such as Big-Five personality traits, one’s first thought might be typical-performance measures, whereas for other attributes, such as intelligence, one’s first thought might be maximum-performance measures. However, could there be a gain to going beyond these first thoughts and exploring ways of combining the two kinds of measures, especially as both kinds of measures seem, at least in some circumstances, to have predictive or concurrent validity and yet to correlate minimally or not at all with each other (Sternberg et al. 2022)?

What one best measures through typical-performance assessments is different from what one measures through maximum-performance assessments. That is why the two kinds of measurements are compatible—because they measure different things. 

Table 1 shows how abilities and attitudes intersect in the manifestation of intelligence as well as creativity and wisdom. 

Typically, psychologists (and others) view intelligence as an ability. Creativity and wisdom, however, are typically viewed as involving some combination of abilities and attitudes. What I am calling “attitudes” here are sometimes referred to as “dispositions” in the literature on critical thinking (e.g., Ennis 2011), but the term “disposition” might suggest a state of mind that is somewhat “baked in,” whereas the states of mind being discussed here are highly modifiable and susceptible to change when people want them to change. 

In terms of abilities, intelligence involves skills such as the ability to acquire knowledge and the ability to think critically or analytically about that knowledge. Creativity involves the abilities to generate novel ideas and to generate ideas that are useful. Wisdom involves the ability to balance one’s own interests with other people’s interests, and to find a common good, not just a personal good or a good for members of one’s perceived tribe. 

Creativity and wisdom typically are viewed as some combination of abilities and attitudes (see essays in Kaufman and Sternberg 2019; Sternberg and Glück 2019). For example, with regard to creativity, one may need to generate ideas that are novel and useful. There are any number of reasons why attitudinally, one might not wish to do so, as discussed in more detail later—for example, there may be extreme social pressure and even legal pressure to conform to whatever the going thinking is. Wisdom, too, requires not only the ability to balance interests and to seek a common good but also the attitude to want to balance interests and seek a common good. Some people have the ability to do these things; they simply decide not to. They put themselves first and perhaps second, third, and last too.

Whereas creativity and wisdom have been conceived as a combination of abilities and attitudes that make the deployment of those abilities possible, intelligence typically has been thought of exclusively or almost exclusively as an ability or set of abilities (see essays in Sternberg 2020c). Why should intelligence be different from creativity and wisdom in being the only one of the “Big Three” to be associated with an underlying set of abilities but not an underlying set of attitudes? I suggest here that it is not different—that attitudes are at least as important for intelligence as they are for creativity and for wisdom and that, beyond that, many of the problems the world faces today stem not from a lack of the abilities of intelligence but rather from a lack of the attitudes that precede and accompany the utilization of the abilities of intelligence.

In the case of intelligence, there are three overarching attitudes that are key. These are attitudes toward the (a) the desire to acquire relevant information, (b) the integrity of the processing of the information, and (c) the positivity toward which the information will be put. I consider each of these attitudes in turn.

First, the desire to acquire relevant information might seem like a given, but it is far from a given. There are multiple reasons why people might *not* choose to acquire relevant information for the tasks that face them. Many people buy into an increasing tendency in the world to rely on *authority* and *faith* rather than *induction* as a means of acquiring information. Increasingly, it seems, countries such as Russia and China that previously had been moving in the direction of democracy have instead moved in the direction of autocracy, where faith in the ultimate authority of a dictator is supposed to dictate how one thinks and acts. Thinking or acting otherwise can come with severe costs to one’s personal safety and even life. In the United States as well, authoritarianism and acceptance of it have been on the rise. 

Second, one’s overarching attitude in using one’s abilities might be referred to as intellectual integrity (see Sternberg 2021b). This is a desire for information that is internally consistent (i.e., it makes sense) and externally correspondent with reality (i.e., it is true). If people do not care if information makes sense, or if it is true, their intelligence will likely be put to suboptimal uses (Sternberg 2022). They may be entrenched in their thinking, or beholden to faith in authority, or simply seeking to use information for their own manipulative purposes.

Third, an overarching attitude is whether people seek to use their intelligence for good or for bad purposes. When information is used to make the world better, on balance, for everyone, not just for oneself, it might be referred to as “adaptive intelligence” (Sternberg 2021a).

Although I have focused on three examples of attitudes of intelligence, there are many others. These include, for example, (a) seeking from others constructive criticism of one’s views; (b) having an open mind; (c) looking to learn from and collaborate with other people; (d) feeling license to change one’s views as time passes; (e) thinking metacognitively—seeking understanding and control of one’s knowledge and thought; (f) being willing to learn from one’s mistakes; (g) seeking to critique one’s own ideas; (h) adapting to alternative contexts and realizing that a response that works in one context does not necessarily work in another; (i) being willing to be flexible in one’s thinking; (j) knowing when and how to shift one’s perspectives; (k) being willing to learn from one’s failures as well as other people’s; (l) figuring out how effectively to learn what one does not know; (m) realizing that one’s knowledge is incomplete and seeking to complete it to the extent possible; (n) welcoming intellectual adversity as a way to grow in one’s thinking; and (o) being willing to overcome obstacles.

Although intelligence has not typically been seen as involving attitudes as well as abilities, critical thinking, a related construct, has been. For example, Ennis (2011) defined critical thinking as “reasonable and reflective thinking focused on deciding what to believe or do.” Some of the attitudes that Ennis identified are related to metacomponential thinking, such as seeking to identify a question and to formulate criteria for judging possible answers. Others also have identified attitudes (also identified as “dispositions” as well as abilities for critical thinking (e.g., Dewey [1938] 1997, [1916] 2010; Dwyer 2019; Halpern 2022; Hitchcock 2018; Lipman 1987; Marr 2022; Paul and Elder 2019).

Halpern and Dunn (2021) refer to “critical thinking” as “intelligence for solving real-world problems,” a view that perfectly fits that of the current argument. In terms of the theory of adaptive intelligence (Sternberg 2021a; see also Sternberg 1985, 1997a), critical thinking as it is typically conceived of and measured combines analytical and practical intelligence. With analytical intelligence, one analyzes, judges, compares and contrasts, critiques, discerns, and evaluates material, but often material that has little or no relevance to the real world. Critical-thinking problems of the kind considered by Halpern and Dunn (2021) and others take analytical processing and apply it not merely to abstract-analytical problems, but also to problems of the kinds found in everyday life. Thus, in general agreement with Halpern and Dunn, critical thinking is indeed intelligence applied to life, although not all intelligence applied to life involves critical thinking. For example, practical intelligence often draws on tacit knowledge of knowing how things work, based on procedural knowledge acquired from experience (Hedlund 2020; Sternberg et al. 2000)—such as of how to write an article for a journal, including this one. We are not at the point yet of having empirical data for a test of intelligence as an attitude. We have a preliminary experimental measure, but it is just starting to be validated and measurement questions will need to be worked out as part of this validation. Examples of items in this preliminary measure are “When I solve life problems, I try to look at those problems from many different points of view” and “I have an intuitive sense of right and wrong that I can trust in virtually any situation” [inverse scored]. 

A given critical-thinking problem will be a mix of practical with analytical. Practical problems differ from IQ-test-like analytical problems in that (a) they have no single correct answer, (b) the nature of the problem changes while one is attempting to solve it, so that the problem one must solve does not remain the same throughout the problem-solving process, (c) they tend to be ill-defined, with no clear path to solution, (d) they are time-consuming, (e) they are emotionally involving and sometimes gut-wrenching, (f) they are often for high stakes, which may even be life-changing, (g) they are solved in groups, (h) they do not provide all the information needed to solve the problem, (i) they are almost never short-answer or multiple-choice, and (j) there often is no one right answer, or they may perhaps lack even any fully satisfactory answer (Sternberg 2020a). 

Critical thinking also should be applied to psychologists’ thinking about psychology (e.g., Gernsbacher et al. 2015; Stanovich 2018; Sternberg and Halpern 2020) and intelligence researchers’ thinking about their own studies of intelligence. Sternberg (1990), for example, points out that although many intelligence researchers are adherents to a psychometric (which he calls a “geographic”) approach to intelligence, there are multiple approaches, such as anthropological, sociological, cognitive, developmental, biological, genetic-epistemological, and systems approaches. Sternberg’s own research has undergone transformation with regard to the approaches he describes: It started out as largely cognitive with some psychometric overtones, then took on developmental aspects, and today would be regarded as systems-based. Whereas Sternberg initially largely rejected the psychometric approach, he now incorporates aspects of it into his own current theory (Sternberg 2021a). 

On this view, then, the attitudes and abilities of critical thinking are not separate from intelligence, but rather, some mix of analytical and practical intelligence. (Some scholars also include creative intelligence as part of critical thinking—it is a matter of definition.) The attitudes are as important as the abilities, because without the attitudes, the intelligence is never brought to bear on the problems that require it, or is brought to bear in a biased way. For example, consider cults, whether of religion (e.g., the apocalyptic cult of Christian Nationalism in the United States right now that, in some cases, distorts history to suit racist and xenophobic goals) or of politics (e.g., the cult of Vladimir Putin or of Donald Trump. The cults invent apocryphal histories to suit their goals of obtaining and staying power. The problem in many of their followers is not lack of intelligence as an ability, but rather, lack of the attitudes of applying analytical thinking to transparently false and often self-contradictory statements. For example, Vladimir Putin has changed his story about the goal of the war in Ukraine multiple times, a fact that does not seem to bother his followers—recently, it is no longer merely about Ukraine not being its own country, but also about a war on the “Satanism” of the West, an odd statement from a leader who, so far with impunity, authorizes the commission of murders (Rettman 2022). How can intelligent people fall for cant such as this? 

IQs rose 30 points in the twentieth century (Flynn 1987, 2012, 2016, 2020), and yet, at times, it is hard to see how that intelligence plays out in real-world events. One possible reason for what often seems like real-world stupidity (Sternberg 2002) is that people who are intelligent also can be foolish, regardless of how smart they are (Aczel 2019). Indeed, being smart may make people more susceptible to being foolish because smart people may think that their intelligence prevents them from thinking and acting in foolish ways (Sternberg 2004). Thus, the people are “intelligent” in a conventional sense but neither wise (Sternberg and Glück 2022) nor adaptively intelligent (Sternberg 2021a). 

Another possible reason people fall for cant, however, is that intelligence rose in levels as an ability but not as an attitude—people have the intelligence; they just do not deploy it effectively, with integrity, or with good intent. Why are so many people losing their lives in the War in Ukraine today? Why do so many people, companies, and even governments actively undermine efforts to control global climate change? Why do people knowingly pollute the environment? There is no question that many of these agents, probably most, know better. They just choose not to use the intelligence they have effectively, or they choose to use it for ends that lack integrity or that are just bad.

## 3. The Twin Challenges of Blind Faith and Reliance on Authority

Why would people choose not to use their intelligence—in other words, why would they have the intellectual ability but not deploy the intellectual attitude fully or sometimes even partially? There are a number of reasons. All of them involve, at some level, faith in authority.

First, people sometimes are intellectually lazy, just as they are sometimes physically lazy. It is easier to rely on faith in authority than to carefully think things through for oneself. If one reads only sources with which one already agrees—a result of the commonly observed myside bias (Stanovich 2021)—one will save oneself intellectual effort at the same time that one believes that one is obtaining independent confirmation of one’s prior beliefs.

Second, one may make an effort, but nevertheless show the same myside bias, consulting only sources with which one has an ideological affinity. The result is the same. One is making an effort but showing a non-intellectual attitude. Instead of seeking multiple points of view, one seeks only the point of view that corresponds to one’s own.

Third, in rigid autocratic societies such as China, Russia, North Korea, and Iran, displaying publicly an intellectual attitude, or even showing it in a way that one thinks is private but that turns out not to be, can be extremely costly to one’s health and safety. In Russia today, the laws allow for 15 years of imprisonment (Mathers 2022) for saying things that are true but inconvenient to the government, such as that the Ukrainian “special military operation” is actually a war, conceived through the whim of a dictator, and imposed by Russia on Ukraine and on the world. Dissidents in Russia also have a way of getting poisoned or falling out of windows of high buildings (King 2020; Rahman 2022).

Fourth, people may be educated in ways that lead them to accept the words of authority on faith. For example, in some religious and ideologically-based schools, children are taught that what their religious or ideological leaders say, goes. The words and deeds of those authorities are not to be questioned. The result is abuse of the students’ minds and often, unfortunately, their bodies.

Fifth, in virtually all societies, there is pressure to conform—to go along with the crowd (Sternberg 2018; Sternberg and Lubart 1995). It is easier to have faith in the authority of the crowd—the view that if a lot of others believe something, it must be true—than to defy the crowd or the societal Zeitgeist and think for oneself.

Finally, and perhaps most important, intelligence provides no protection against evil, and may even be used in the service of evil. Whereas adaptive intelligence is defined as intelligence used in the service of a common good, general intelligence has no such constraint. It can as easily be used for evil ends as for good ones. Some of what seems “stupid” to us may be actions that are immoral. Because we cannot readily accept that smart people would quickly do evil things, we may write their evil actions off as temporary stupidity when they are anything but. A lot of IQ points have gone into the perpetration of the genocidal war in Ukraine, for example. The fundamental principle of interpersonal attraction is that like attracts like (Sternberg 1998): People who smart but evil who get into power look for others like themselves to support their policies and to execute those policies. Invariably, they find sycophants to do their “dirty work”.

## 4. Relation to Other Constructs

The idea of intelligence as an attitude is related to other constructs explored by previous theory and research. Some of these have been alluded to in the article. They are considered here in more detail. In particular, consider dispositions of critical thinking, personality traits such as openness, wisdom, and cognitive (thinking) styles.

### 4.1. Dispositions of Critical Thinking

Intelligent attitudes are related to critical-thinking dispositions, as proposed by Ennis (2011), among others cited earlier. Examples of critical-thinking dispositions are that thinkers care that their beliefs are true, seek alternative hypotheses, try to be well-informed, and endorse a position only to the extent that it is justified by the [true] information that is available. 

Oxford Languages (n.d.b) defines a disposition as “a person’s inherent qualities of mind and character”. Synonyms are “temperament, nature, character, constitution, makeup, grain”. The term “disposition” has a long history in philosophy, way preceding its use in psychology. The *Stanford Encyclopedia of Philosophy* (n.d.) notes that many philosophers view dispositions as intrinsic to their bearers. A disposition can also be an inclination or tendency. 

In contrast, Oxford Languages (n.d.a) defines an attitude as “a settled way of thinking or feeling about someone or something, typically one that is reflected in a person’s behavior” with synonyms of “point of view, view, viewpoint, frame of mind”. (I earlier defined an “attitude” as a developed mindset or approach toward something that is capable of change.).

To the extent that a disposition is of nature, character, or constitution, it is quite different from an attitude. An attitude is modifiable and not inherent. The goal here is not to split hairs over definitions. It is to point out that the current view is similar to that of critical-thinking dispositions but probably not the same. What distinguishes an attitude is, at minimum, that it is (a) situational, (b) a function of the task at hand, and (c) modifiable and potentially flexible. It is not constitutional or characterological but rather a frame of mind one can adopt or discard at will.

### 4.2. Personality

Intelligence as, in part, an attitude, bears some relationship to personality. Early investigators investigated the relationship between personality and persuasibility (Hovland and Janis 1959). They found that three ability factors—the abilities to attend to information, comprehend information, and anticipate information--were positively related to persuasibility, but that the ability to evaluate the information was negatively related to persuasibility. More persuasible people, in other words, are less evaluative of information.

Intelligence is related to the construct of openness to experience in the five-factor theory of personality (e.g., McCrae and Costa 1997, 2003, 2008) and probably bears a relationship to conscientiousness as well. The other factors of the five-factor theory are extraversion, agreeableness, and neuroticism. which are probably less closely related to intelligence as an attitude. The five-factor theory is perhaps the most widely accepted among personality theorists. A related theory, HEXACO, posits six basic traits: Honesty-Humility, Emotionality, eXtraversion, Agreeableness (versus Anger), Conscientiousness, and Openness to Experience (Ashton and Lee 2007; Lee and Ashton 2018). This theory also is in widespread use, with openness again related to intelligence.

In one version of the five-factor theory, openness to experience has six correlated facets (Costa and McCrae 1992): The first is active imagination and fantasy, which is a well-developed fantasy life. The second is aesthetic sensitivity, as in one’s the ability to appreciate beauty and excellence in artistic or musical compositions. The third is attentiveness to feelings, or one’s attention to but also insights into how one feels about things. The fourth is preference for variety, or adventurousness and avoidance of sameness. The fifth is intellectual curiosity, or one’s willingness to learn about new things and become interested in them. Additionally, the sixth is challenging of authority, which is often associated with psychological liberalism. Liberalism is not necessary a matter here of ideology but rather of liking to think in new and different ways (Sternberg 1997b).

Openness and intellectuality sometimes are viewed as of one piece, as a personality trait that can be labeled openness/intellectuality (Oleynick et al. 2017). A somewhat different version of the model has separated openness from intellectuality. Indeed, these two constructs have been found to predict somewhat different abilities, for example, with openness predicting creativity and intellectuality predicting fluid intelligence (Nusbaum and Silvia 2011).

Two personality theorists, Murray (1938) and later McClelland (1988), proposed a theory of needs, such as needs for achievement, affiliation, and power. Another relevant personality construct is need for cognition (Cacioppo and Petty 1982; Cacioppo et al. 1996). Need for cognition is a penchant for engaging in intellectual activity. It is related negatively but weakly to closed-mindedness but is positively related to intelligence. 

Another related construct is credulity, or a tendency to believe what one reads or hears without much regard to its credibility (Sternberg forthcoming). People differ in the extent to which they are willing or able to assess source credibility, so that for some people, authority is good enough, regardless of its credibility. True Believers (Dennett 1981)—people who are totally taken in by the words of an authority figure—are very high in credulity. They completely and usually unreflectively buy into an existing dogma or belief structure as presented by one or more authority figures.

Intelligent attitudes are related to, but rather different from intelligent dispositions or traits. Some of the differences are summarized in Table 2.

In particular, first, traits are relatively task-independent. If, for example, one is extroverted as a trait, the trait tends to permeate one’s behavior, although not necessarily entirely. Attitudes, in contrast, are task-dependent. One might treat work tasks and personal tasks very differently. For example, one might decide to evaluate the credibility of sources in one’s work, but in one’s personal life, gleefully accept gossip about supposed failings in people’s personal lives without checking whether the gossip is true. 

Second, contexts and situations also matter more for attitudes than for fixed traits. In one’s personal life, one might decide to employ critical thinking in situations in which one hears new information from strangers or people whom one does not know well, but to accept without further thought or verification information one hears from people one knows well. Fixed traits are, at least in theory, part of a person’s internal makeup. Attitudes vary across contexts.

Third, one does not have much conscious control over traits. If one is neurotic, for example, it is challenging just to “turn off” one’s neuroticism. In contrast, one decides upon one’s attitudes. They may be influenced by many factors, but in the end, they represent voluntary choices. One generally does not “choose” to be agreeable or neurotic, but one does choose to seek out multiple sources of information and to verify the validity of those sources.

Fourth, attitudes, unlike dispositions or personality traits, are rather readily modifiable. For example, one can decide to vote for a conservative or liberal political candidate at the touch of a button. However, to the extent that one’s choice is motivated by a personality trait, such as openness to experience, the trait itself may be quite a bit less modifiable. For example, if one has never voted for a liberal (or conservative) candidate, it may be hard to change if one lacks openness and finds it hard to vote in a new way because of the newness of the experience, which seems uncomfortable and perhaps unpleasant.

Fifth and sixth, attitudes are highly susceptible to both (a) external and (b) internal influences. Politicians, for example, try to change people’s attitudes toward information by appealing to their emotions: If one can arouse them enough, perhaps they will not think through whether what the politician says makes sense and whether it is worth even paying attention to. In particular, political leaders and other leaders may try to arouse emotions such as fear and anger to convince people to vote for them. The leaders are much less likely, however, to change the people’s fundamental personality traits. Indeed, they may direct their appeal to people whom they believe exhibit certain personality traits or dispositions, such as a tendency toward paranoia or extreme self-absorption.

Seventh, attitudes are less temporally stable than personality traits or dispositions. In times of upheaval due to perceptions of a failed government, even those who once supported the existing government may question their attitudes toward it and, equally, the reasons why they supported it and should continue to support it. They may seek out information that in the past they had taken for granted. Their attitudes thus may change with the times, even while their personality does not.

Eighth and finally, theory and research on traits and dispositions versus attitudes have emanated from somewhat distinct literatures. Research on traits and dispositions has emanated largely from the personality and philosophy literatures, some of which more emphasize the roles of inherent characteristics of persons. In contrast, research on attitudes more has emanated from the social-psychological literature, which more emphasizes the role of situations.

### 4.3. Wisdom

Wisdom has been defined in so many ways that it is difficult to pin down exactly what it is. A recent proposed common-consensus view is that wisdom represents a balance of viewpoints, epistemic humility, context adaptability, and multiple perspectives (Grossmann et al. 2020). Sternberg (1998, 2019) has proposed a balance theory of wisdom, according to which wisdom involves seeking a common good; by balancing intrapersonal (one’s own), interpersonal (others’), and larger interests; over the long- as well as the short-term; through the infusion of positive ethical values; by adapting to, shaping, and selecting environments. On this view, people who are wise know what they know, but also what they do not know and, at the same time, what they cannot know (i.e., what is currently unknowable—see also Swartwood and Tiberius 2019). Glück and Weststrate (2022) have proposed that in challenging everyday situations, noncognitive components of wisdom (in particular, an exploratory orientation, concern for others, and emotion regulation) moderate the effect of cognitive components (in particular, knowledge, metacognitive capacities, and self-reflection) on wise behavior.

The current view on intelligent attitudes overlaps with views of wisdom because most models of wisdom, unlike dominant contemporary models of intelligence, include attitudinal as well as ability-based components. The Glück and Weststrate (2022) model specifically views attitudes as well as dispositions as moderating effects of cognitive processes. What is different is that wisdom necessarily must be conceived of as partially attitudinal, because it involves judgments about personal or societal values, achieving a common good in particular situations, and resolving specific uncertainties in life; attitudes have not been part of models of intelligence, at least dominant ones, on the other hand, because many problems can be solved (e.g., vocabulary items, deductive-reasoning problems, or spatial-rotation problems) without applying personal or other values. 

Attitudes *are* important for intelligence, however, because they determine, to a large extent, whether one will choose to solve the problems one confronts in a more or less intelligent way, or without the application of intelligence at all. Or one’s attitudes may lead one to apply one’s intelligence, but only to support a prior viewpoint. Adaptive intelligence, like wisdom, involves seeking a common good, but on the scale of what is biologically adaptive for survival of a gene pool or of humanity in general, not just in judgments of everyday matters (Sternberg 2021a).

### 4.4. Cognitive/Thinking/Intellectual Styles

There are a number of different terms for related stylistic constructs—cognitive styles, thinking styles, intellectual styles—that are, in large part, different names for similar phenomena, namely, preferences for how people choose to use their abilities (Sternberg 1997b; Witkin and Goodenough 1981; Zhang et al. 2012). Styles are measured in one of two ways. 

Typical-performance tests ask participants about their preferred types of problems or ways of solving problems. Three examples of styles measured through typical-performance tests are the legislative, executive, and judicial styles in Sternberg’s (1988, 1997b) theory of mental self-government. People with a legislative style like to choose things to do, to do them their own way, and to structure their own problem-solving experiences. People with an executive style prefer to be given more structure, and often just to be told what to do and how to do it. People with a judicial style prefer to judge the work of others, analyzing and critiquing it for its strengths and weaknesses. An example of a legislative item might be “I like to do things my own way”. An example of an executive item might be “I prefer tasks that have a clear structure”. An example of a judicial item might be “I like to judge the work of others”. The applicability of the statements would be rated on a Likert scale. Some of these styles generally correlate with measured intelligence (Zhang and Sternberg 2006).

Maximum-performance tests require participants to solve challenging cognitively based problems, such as distinguishing an object from its surrounding context. An example of a cognitive style measured in this way is field independence versus field dependence (Witkin 1950; Witkin and Goodenough 1977, 1981; Witkin et al. 1962, 1977). A person who tends to be field independent consistently refers to and relies heavily on internal referents in problem solving. Such referents might include bodily sensation cues or simply metacognitive cues regarding how to process information. A person who tends to be field dependent relies more on external referents (environmental cues and surrounding contexts). A test of field independence might involve recognizing particular figures embedded in a larger context, or orienting one’s body upright with respect to the ground despite confusing cues from the surrounding context. (This is a task that a pilot of a plane would confront in the absence of instruments specifying orientation. Cues, especially on cloudy or hazy days, can be so confusing that sometimes pilots believe their intuitions rather than their instruments, generally to their detriment.) Field independence, although it is supposed to be a cognitive style, correlates significantly and consistently with spatial ability (e.g., Boccia et al. 2016). A reflective cognitive style also predicts intelligence-test performance (Alaybeck et al. 2021).

Thinking (cognitive, intellectual, and related) styles can overlap with intelligent attitudes, although the styles tend often to be more ingrained. For example, being reflective about problems that require deep thought is an intelligent attitude. However, the overlap is, at best, partial, because thinking styles are of so many different kinds (Kozhevnikov et al. 2014; Sternberg et al. 2008; Zhang et al. 2012). In general, though, they are preferences that can lead to better intellectual performance or worse intellectual performance. For example, being impulsive in solving a problem that requires reflective thought potentially will result in a solution that is not adequately thought through; being field-dependent may be harmful in spatial tasks. Thus, whereas intelligent attitudes lead to better intellectual performance, cognitive and thinking styles may lead to intellectual performance that is better or worse or may have no relation at all to intellectual performance.

## 5. Conclusions

Creativity and wisdom have long been seen as having necessary attitudinal as well as ability-based components. I have argued in this article that intelligence does as well. We ignore the attitudinal-based component at our own peril. Many of the problems we face in the world stem not from a lack of intelligence as an ability, but rather from a lack of an intelligent attitude. People choose not to use, or to use in a biased way, or not to use much of the intelligence they have. 

If we intend on developing intelligence, we must develop intelligent attitudes, just as we need to develop critical-thinking dispositions (Ennis 2011; Halpern 2022; Halpern and Dunn 2021; Paul and Elder 2019), wisdom (Sternberg and Glück 2019), creativity (Kaufman and Sternberg 2019), and intelligence as an ability (Sternberg 2020c). By focusing only on the ability-based component of intelligence, society has rewarded people who can, in principle, do various things, not those who have the attitude to do them, in practice. It is like rewarding someone with a great deal of musical talent who perhaps chooses never to play an instrument, compose, sing, or otherwise put their musical talent into practice. 

We need to develop not just the ability-based component of intelligence, but also the attitudinally based one. We also need to develop measures of this attitudinally based component, keeping in mind that such attitudes are modifiable and should be modified through schooling. Given the challenges facing the world, it is not enough to view intelligence merely as an ability—we need to view it as having an attitudinal component, without which the ability may rarely be used as it could and should be.

There are steps educators could take right now. First, we need to develop assessments that measure intelligence as an attitude, not just as an ability, so that schools will pay attention. Some tests of the related construct of critical thinking already exist (e.g., Ennis 1993, 2005; Facione 1990, 1992; Halpern 2010; Watson and Glaser 1980). So, the field has a start that could be further integrated with the assessment of intelligence. We are ourselves researching at this time a measure of intelligent attitudes. Second, we should develop curricula that encourage and augment intelligence as an attitude. Third, we need to revise theories of intelligence to include intelligence as an attitude, not just as an ability. Fourth, we should have an intelligent attitude ourselves, so that rather than remaining entrenched in current views of intelligence, we open ourselves to realizing that current conceptions of intelligence simply as an ability fail to account for why intelligence is so often poorly used, if it is used at all.

## Figures and Tables

**Table 1:** Ability-Based and Attitudinal Aspects of Intelligence, Creativity, and Wisdom.

*Characteristic/Aspect*	Ability	Attitude
**Intelligence**	Skill in acquisition of knowledge Skill in critical thinking with (analysis of) that knowledge	Deciding to acquire knowledge Deciding to think critically with the knowledge one acquires
**Creativity**	Skill in generation of novel ideas (divergent thinking) Skill in generation of useful ideas	Deciding to seek ideas that are divergent from those of others Deciding to seek ideas that someone will find useful
**Wisdom**	Skill in balancing one’s own interests with others’ interests Skill in finding a common good	Deciding to seek to balance one’s own interests with those of others Deciding to seek a common good

**Table 2:** How Attitudes Regarding Deployment of Intelligence Differ from Inherent Dispositions and Personality Traits.

*Type of Characteristic/* *Source of Difference*	Intelligence-Related Dispositions/Personality Traits	Intelligence-Related Attitudes
**Tasks**	Task-Independent	Task-Dependent
***Contexts/Situations***	**Contextually/Situationally General**	**Contextually/Situationally** **Bound**
**Level of Conscious Control**	Largely Uncontrollable	Largely Controllable
**Modifiability**	Largely Unmodifiable	Largely Modifiable
**Susceptibility to External Influence**	Largely Unsusceptible to External Influence (e.g., Authority Figures, Peer Groups)	Highly Susceptible to External Influence (e.g., Authority Figures, Peer Groups)
**Susceptibility to Internal Influence**	Largely Unsusceptible to Internal Influence (e.g., Mood, Emotions)	Highly Susceptible to Internal Influence (e.g., Mood, Emotions)
**Temporal Stability**	Temporally Relatively Stable	Temporally Relatively Labile
**Most Relevant Past Research**	Personality Psychology (also certain Schools of Philosophy)	Social Psychology

## Data Availability

This article reported no new data.
